# Mitochondrial DNA variation, genetic structure and demographic history of Iranian populations

**Published:** 2014-03

**Authors:** Fatah Zarei, Hiva Alipanah

**Affiliations:** Department of Zoology, Faculty of Biological Sciences, Shahid Beheshti University, Tehran, Iran

**Keywords:** Iranians, mtDNA, Haplogroups, Evolutionary history

## Abstract

In order to survey the evolutionary history and impact of historical events on the genetic structure of Iranian people, the HV2 region of 141 mtDNA sequences related to six Iranian populations were analyzed. Slight and non-significant F_ST_ distances among the Central-western Persian speaking populations of Iran testify to the common origin of these populations from one proto-population. Mismatch distribution suggests that this proto-Iranian population started to colonize Iran about 30000 years ago which is almost consistent with the timing of arrival and colonization of western Asia by the anatomically modern human. Star-like haplotype network structures, significant and negative Tajima’s D (D=-2.08, P<0.05) and unimodal mismatch distributions support the genetic effects of this expansion. Iranian populations presented mtDNA lineages that clearly belong to the European gene pool (i.e. H and U), while the Mashhad population was characterized by the presence of eastern and central Asian mtDNA lineages (i.e. M, B and D). Furthermore, the low diversity (*h*=0.428) observed in Mashhad may indicated the presence of inbreeding, drift or bottleneck events. The application of Monmonier’s maximum differences algorithm revealed a geographic zone of genetic discontinuity between the Arab people of Khuzestan and rest of Iranian populations. Geographical factors, in cooperation with cultural/linguistic differences, are the main reasons for this differentiation. The lack of a sharp geographical or ethno-linguistic structure for mtDNA HV2 sequence diversity was statistically supported by AMOVA and Mantel (r=0.19, P<0.05) tests.

## INTRODUCTION

Present-day Iran has played a key role in the distribution of the modern human, and has acted as a corridor and natural inter-continental passageway for the expansion of genes [1]. The Neolithic and Metal Ages seem to be the time windows that left the deepest imprint on Iran’s genetic landscape [2]. Currently, two major theories, inspired from archeological, linguistic and genetic evidences, compete regarding the origin of Indo-Europeans as well as the early migration of these people and the colonization of Europe and western Asia [3]. The “Steppe Hypothesis” proposes that early Indo-Europeans entered southeastern Europe from the Pontic Steppes in three waves between 4400 B.C and 2800 B.C [4-7]. Subsequently, another expansion began towards the southeast around 1500 B.C. These people replaced those who spoke the Dravidian language, which was, in turn, almost completely replaced around 1300 B.C by the Indo-Iranian branch of the Indo-European language family upon the arrival of the Aryans who were nomadic tribes currently considered as plausible ancestors of most of the contemporary Iranian people [8]. The “Anatolian Hypothesis”, on the other hand, suggests that Indo-European languages spread with the expansion of agriculture from Anatolia, beginning from 9500 to 8000 years ago [9]. Genetic evidence from present-day populations supports this hypothesis [10-13].

The analysis of DNA samples provides a powerful tool for the reconstruction of evolutionary history in extinct and extant species [14, 15]. Reconstructing demographic history allows us to gain useful insights into different evolutionary processes by evaluating correlations between demographic and Palaeoclimatic events [14, 16], testing the elements driving past population dynamics [17-19], and tracing the transmission and expansion of viruses [20, 21]. In the past two decades, genetic markers have been widely used to infer the origin, migration and admixture of human populations. Among them, mtDNA and the non-recombining portion of the Y-chromosome have been shown to be more informative in tracing human evolutionary history since they only transmit through maternal and paternal lineages, respectively. For mtDNA markers, the HV segment in the D-loop region has a higher mutation rate than the rest of the mtDNA, and has therefore become the most studied marker used for inferring genetic relationships among different populations with a plethora of data from worldwide populations for comparison [22-25].

Despite the fact that the Iranian corridor still hosts and is surrounded by populations with very different backgrounds in terms of origins, languages, religions and modes of subsistence, the genetic diversity of very few Iranian ethnic groups has been investigated so far [26-31]. These studies mostly targeted a vast geographic scale and more general questions about the genetic relationships between different populations. Estimating the effects of neighboring mtDNA pools on the genetic landscape of present-day Iranian people is a crucial matter. Thus, in order to investigate the origin, genetic structure, and the genetic relationships between Iranian populations, the present study provides data on mtDNA variations in 141 individuals from six Iranian populations with different linguistic and geographic origins. For inter-population comparisons and estimation of the effects of neighboring ethnic groups on Iranian people’s genetic structures, sets of the HV2 nucleotide sequences were obtained for regional groups of populations from adjacent regions to address the following questions: (1) How genetically close are the Iranian populations living in different areas and/or speaking different languages? (2) What is the genetic relationship between Iranian Indo-European speaking groups and other neighboring Indo-European and non-Indo-European populations? (3) Is there any specific linguistic or geographic structure governing the mtDNA diversity? (4) Can a source population for the Persian speaking populations from Iran be identified? (5) Is there any genomic boundaries between the Iranian populations? and (6) Which of the evolutionary forces been involved in shaping the genetic landscape of present-day Iranians. The comprehension of this specific case study can help clarify the genetic structure and origin of Iranian populations. 

## MATERIALS AND METHODS

**Figure 1:**
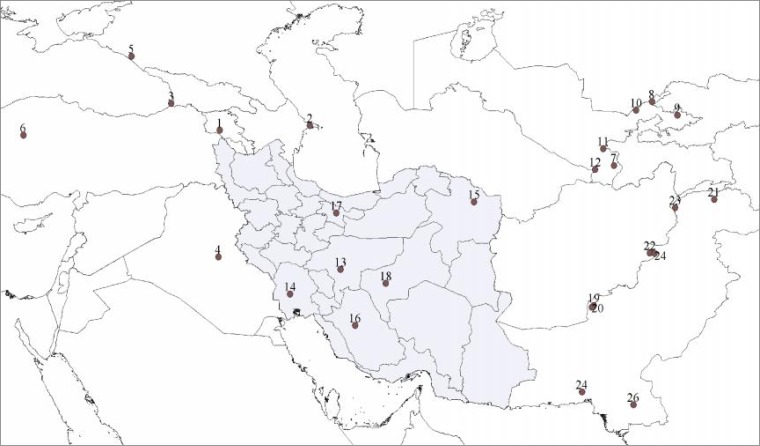
Geographic distribution of 26 populations presented in this study

**Study populations:** HV2 nucleotide sequences of 141 individuals from six Iranian populations including five Persian speaking populations from Tehran [32], Esfahan, Yazd, Shiraz, Mashhad, and one Arab population from Khuzestan province (GenBank Accession Numbers EU239536 to EU239655) were obtained from the GeneBank database (Table 1). For inter-population comparison purposes and estimate the effects of neighboring ethnic group’s mtDNA pools on the Iranians genetic landscape, sets of the HV2 nucleotide sequences were obtained from three regional groups of populations (Fig. 1). These were populations from (1) Central Asia [33] including Kazakhs, Kirgizes, Tajiks, Turkmens, Afghans and Russians; (2) Pakistan including Baloches, Brahuis, Burushos, Hazaras, Kalashes, Makranis, Pathans and Sindhis (GenBank Accession Numbers EU565766 to EU566829); and (3) the Anatolia/Caucasus region including Armenians, Georgians, Azeris, Turks [32], Iraqi Kurds [34] and Adygei people of the Caucasus region.

**Table 1:** Geographic localities, coordinates, basic parameters of molecular diversity and Neutrality test results for all populations

Po. €	Populations	Longitude	Latitude	N	s	H	h	π	*k*	Tajima's D (p)
1	Armenian	44.51	40.18	30	15	8	0.682	0.006	1.84	-1.81^*^
2	Azeri	49.86	40.43	30	14	8	0.510	0.004	1.42	-2.00^*^
3	Georgian	41.63	41.63	28	27	11	0.793	0.011	3.32	-1.89^*^
4	Kurds-Iraq	44.42	33.32	15	4	3	0.600	0.004	1.37	0.34(0.67)
5	Russ-Caucasus	39.26	44.17	17	10	6	0.742	0.007	2.18	-1.06(0.15)
6	Turkey	32.85	39.92	29	22	12	0.800	0.008	2.37	-2.00^*^
7	Afghan	67.89	38.27	98	21	10	0.574	0.005	1.67	-1.57 ^*^
8	Kazakh	70.15	41.73	247	58	40	0.645	0.008	2.44	-2.18 ^*^
9	Kirgiz	71.66	41.00	226	42	27	0.504	0.005	1.72	-2.25 ^*^
10	Russ-Central Asia	69.21	41.26	151	35	24	0.718	0.008	2.49	-1.98 ^*^
11	Tajik	67.26	39.18	234	42	27	0.598	0.009	2.73	-1.93 ^*^
12	Turkmen	66.78	38.06	248	48	30	0.677	0.007	2.37	-2.05 ^*^
13	Esfahan	51.67	32.65	23	17	9	0.822	0.011	3.39	-1.18(0.11)
14	Arabs from Khuzestan	48.68	31.31	23	27	12	0.932	0.017	5.25	-1.38(0.07)
15	Mashhad	59.60	36.30	21	18	6	0.428	0.006	2.04	-2.24 ^*^
16	Shiraz	52.53	29.61	23	22	11	0.806	0.011	3.42	-1.82 ^*^
17	Tehran	51.42	35.69	29	23	13	0.854	0.011	3.43	-1.50 ^*^
18	Yazd	54.36	31.89	22	22	11	0.792	0.010	3.25	-2.01 ^*^
19	Baloch	66.69	30.70	25	19	9	0.756	0.011	3.48	-1.52 ^*^
20	Brahui	66.57	30.60	25	11	6	0.566	0.005	1.50	-1.70^*^
21	Burusho	73.84	36.44	25	15	7	0.633	0.009	2.76	-0.85(0.23)
22	Hazara	70.02	33.53	25	6	4	0.466	0.003	1.16	-0.86(0.25)
23	Kalash	71.53	35.98	25	10	5	0.756	0.011	3.44	0.57(0.77)
24	Makrani	66.00	25.99	25	18	8	0.690	0.008	2.58	-1.84 ^*^
25	Pathan	70.31	33.52	25	16	8	0.636	0.007	2.29	-1.85 ^*^
26	Sindhi	69.04	25.30	25	28	10	0.690	0.016	4.78	-1.42(0.08)
-	Iran(total)	-	-	141	51	34	0.810	0.008	2.69	-2.08 ^*^
-	total	-	-	1694	106	125	0.673	0.008	2.51	-2.24 ^*^

* p< 0.05

€ Population number on map; sample size (n), number of polymorphic sites(s), number of haplotypes (H), mean number of nucleotide differences (*k*), haplotype diversity (*h*), nucleotide diversity (π).

**Sequence alignment:** Sequence alignment was first performed using the ClustalW procedure implemented in Mega, version 5.2, and then by hand [35].

**Statistical analysis: **Basic parameters of molecular diversity such as the number of haplotypes (H), the number of polymorphic sites (s), the mean number of nucleotide differences (*k*) [36], and nucleotide (π) and haplotype (*h*) diversity [37] were calculated for each population using Arlequin package version 3.5 [38]. Mega version 5.2, was used to align HV2 sequences to the revised Cambridge reference sequence (rCRS) [39] and detect the polymorphic sites. mtDNA haplogroups were determined based on diagnostic sites in the HV2 region following the mtDNA tree Build 15 (http://www.phylotree.org/) [40]. Evolutionary relationships of the observed mtDNA haplotypes were displayed by a phylogenetic method known as NeighborNet [41] using the SplitsTree version 4 software package [42]. Thus, SplitsTree was employed to build a split network depicting the proximity among haplotypes in a non-dichotomous fashion, with the uncorrected P, NeighborNet distance and Equal Angle algorithm methods (default options). The advantage of this type of cluster analysis is that it allows the cycles or reticulations within evolutionary pathways to accommodate the elevated mutation rates and the corresponding homoplasy of particular genetic systems [43-45]. 

The best probabilistic model of sequence evolution was determined using the software JModeltest version 2.1.3 [46] and the Akaike information criterion. Pairwise F_ST_ genetic distance values were calculated based on the number of pairwise differences between sequences and the K2P model of nucleotide substitutions. The statistical significance of pairwise F_ST_ genetic distances was estimated by permutation analysis using 10000 random permutations. These values were used to evaluate the genetic differentiation of different populations. A neighbor-joining tree [47] was built from the F_ST _distance matrix. The distance matrix was also represented by non-linear multidimensional scaling (NM-MDS) using the STATISTICA 10 package (StatSoft Inc.) [48].

 Changes of effective population size through time were examined following two different approaches; 1) a neutrality test against population growth and 2) the distribution of pairwise differences (mismatch distribution or MMD). First, potential departures from a null hypothesis of the mutation-drift equilibrium and constant population size were estimated by computing the Tajima’s D test for selective neutrality [49]. Thus, negative values of Tajima’s D statistic could reveal recent demographic expansions. Second, we analyzed the distribution of all pairwise haplotype differences and calculated the goodness-of-fit of the estimated distribution to that predicted by a sudden expansion model using 1000 computer simulations [50]. Mismatch distributions were graphically displayed in Microsoft Excel 2007. Mismatch distributions tend to be unimodal, and smooth (i.e. wave-like) in populations that have undergone population size changes. Multimodal or random and rough distributions are characteristics of populations that have experienced long-term stability [51, 52]. The significance or goodness-of-fit of the observed data to the predicted distribution modeled for sudden expansion growth was assessed by using a sum of squares (SSD) method and raggedness index (*rg*) [53, 54]. Significant differences in the sum of the square deviations (P_SSD_<0.05) and raggedness index (P_rg_<0.05) between the observed and simulated mismatch distributions indicated that the population was at a mutation-drift equilibrium (i.e. in a non-expansion phase) [51, 52]. When observed distributions fit the sudden expansion model (P_SSD_≥0.05) using Arlequin version 3.5, the time since the beginning of the expansion (*t*) was estimated from the peak of the distribution (i.e. *τ*) as *t *= *τ*/2μ [55], where μ is the rate of mutation per site per million years multiplied by sequence length. 

Based on a Delaunay triangulation connectivity network, Monmonier’s maximum-difference algorithm [56-58] was used to identify genetic boundaries, namely, geographic zones where differences between populations were largest. The algorithm was applied using the Barrier 2.2 program [59]. To identify groups of neighboring populations with maximum genetic differentiation**,** algorithmic analysis of molecular variance (AMOVA) was applied to the groups classified according to their geographic and linguistic affiliation. This test calculates fixation indices (i.e. Φ_ST_, Φ_SC_ and Φ_CT_) [60], analogous to Wright’s F-statistics [61], allowing the researchers to investigate hierarchical population structure by differentiating variation between groups versus variation within each group. Significance levels of genetic variance components as well as Φ values were evaluated by using 1000 permutations. Eventually, the statistical significance of the correlation between geographic and F_ST_ genetic distance matrices was evaluated by the Mantel test [62] with 1000 permutations using the R vegan library [63]. The Geographic Distance Matrix Generator software, version 1.2, was used to make a geographic distance matrix [64].

## RESULTS

**Figure 2:**
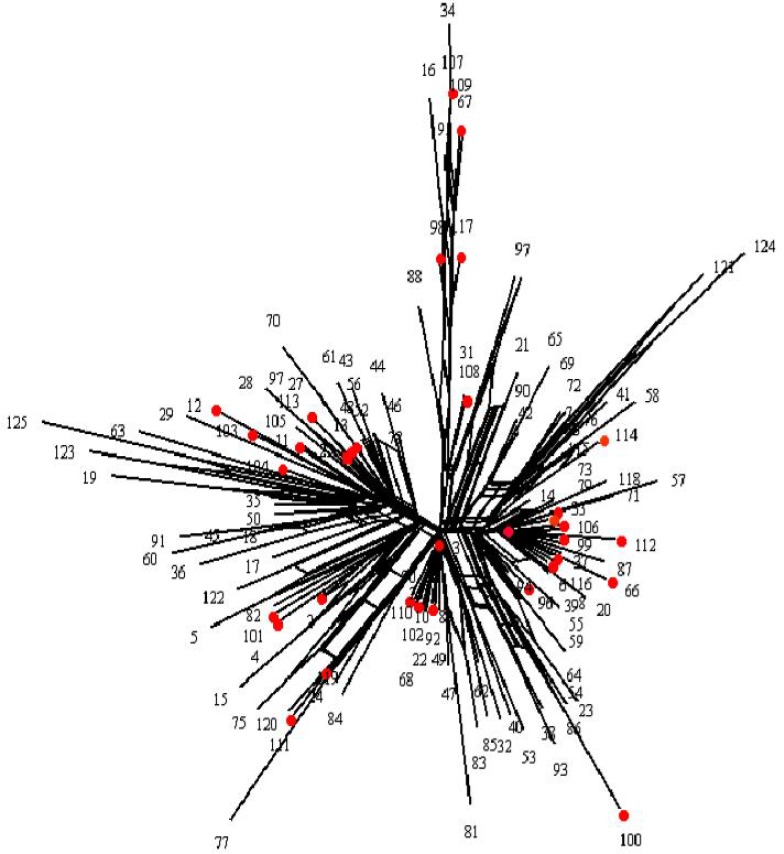
NeighbourNet network for 125 mtDNA haplotypes observed in the all populations. The red circles indicate the haplotypes which observed in the Iranians

**Genetic diversity:** Using 294 bp long sequences comprising nucleotide positions 48 to 342 of the mtDNA control region, we recognized 125 haplotypes in 1694 individuals, which 34 of them observed in the Iranians (Table 1). In addition, 22 out of 34 haplotypes (64.7%) were singletons and only 12 (35.29%) were shared between Iranians. Haplotypes no.3 and no.1 showed the highest frequencies in Iranians (in 56 and 23 individuals, respectively). The unrooted SplitsTree NeighbourNet network in Figure 2 provides a graphic representation of the groups of haplotypes which is not purely dichotomous. Reticulation indicates alternative mutational pathways (i.e. homoplasy) that occur mostly inside each group, as is often the case with D-loop sequences. This allowed us to assign each Iranian haplotype to one of the haplogroups identified. Some parameters characterizing within-population diversity of the mtDNA sequences, such as sample size (n), number of polymorphic sites (s), number of haplotypes (H), haplotype diversity (*h*), nucleotide diversity (π), and mean number of pairwise differences (*k*) are listed in Table 1. Global haplotype diversity was found to be 0.673, ranging from 0.932 for Arab people of Khuzestan province, to 0.428 for Mashhad. Other Iranian populations presented sequence diversities of 0.854 (Tehran) to 0.792 (Yazd). The Central Asian populations presented sequence diversities of 0.718 (Russians) to 0.504 (Kyrgizes). Pakistanians exhibited mtDNA sequence diversities ranging from 0.756 (Kalashes and Baluches) to 0.466 (Hazaras); and the sequence diversity range in the Caucasus region was found to be from 0.8 (Turks) to 0.51 (Azeris). Nucleotide diversity ranged from 0.017 for Arab people of Khuzestan province, to 0.003 for those from Hazara and 0.004 for the Iraqi Kurds (Table 2). In addition, the high and low diversities observed in Khuzestan province and Mashhad were clearly evident in high and low levels of the mean number of pairwise differences (5.25 and 2.047, respectively). The low diversity observed in Mashhad and the Iraqi Kurds may testify to the presence of evolutionary forces such as inbreeding, drift or bottleneck events. 

**Table 2:** MtDNA haplogroups frequencies in the Iranian populations

**Populations**	**L2'3'4+**	**L2a**	**H**	**HV**	**N**	**U**	**R**	**I**	**J1**	**L3**	**T**	**M**	**X**	**B**	**W**	**F**	**D**	**P**	**G**
Esfahan (N=23)	-	-	9	1	2	3	1	1	3	1	1	1	-	-	-	-	-	-	-
Khuzestan Arabs (N=23)	1	-	6	-	2	3	2	-	3	2	-	1	3	-	-	-	-	-	-
Shiraz (N=23)	-	-	5	1	3	4	-	-	2	-	4	2	-	-	2	-	-	-	-
Yazd (N=22)	-	-	7	1	-	3	-	-	1	-	6	-	-	2	2	-	-	-	-
Mashhad (N=21)	-	-	3	2	-	2	-	-	1	-	3	3	1	1	2	1	2	-	-
Tehran (N=29)	-	1	9	1	-	3	-	3	4	-	2	3	-	-	1	-	-	1	1
Total (N=141)	1	1	39	6	7	18	3	4	14	3	16	10	4	3	7	1	2	1	1

**Figure 3:**
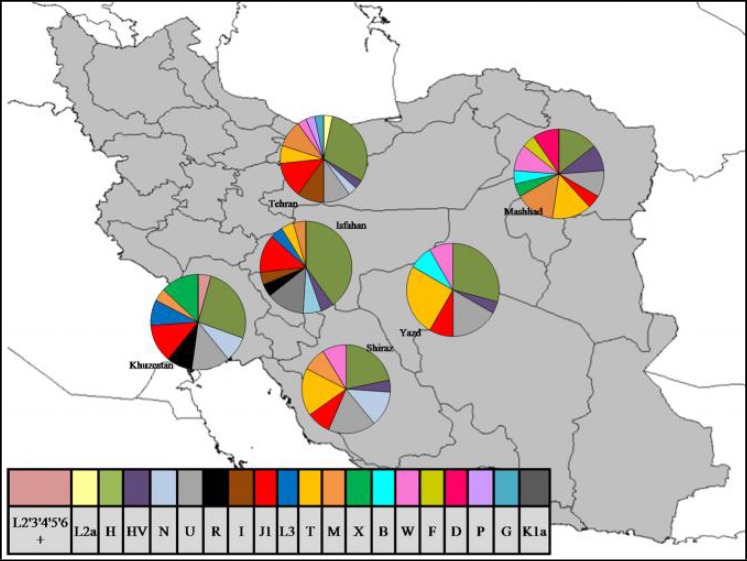
Distribution of the mtDNA haplogroups in the six Iranian populations

**Haplogroup definition:** Each mtDNA haplotype was assigned to a particular mitochondrial haplogroup on the basis of mtDNA TreeBuild, version 15, of the PhyloTree. The frequency distribution of the mtDNA haplogroups was inferred from data on HV2 nucleotide sequences. Data on the distribution of mtDNA haplogroups in all Iranian populations under study are summarized in Table 2. Figure 3 depicts the geographic distribution of the observed mtDNA haplogroups. The mtDNA pools of all Iranian populations were characterized by the presence of European mtDNA haplogroups H and U (at the frequencies of 27.65% and 12.76%, respectively). Other common haplogroups observed were T (11.34%) and J1 (9.92%). Apparently, these mtDNA haplogroups are hardly suitable for studying inter-population relationships and only testify to the common origin of Iranian populations from one proto-population. The distribution of rare or unique haplogroups in populations is more informative. Approximately 58% of all common mtDNA haplogroups (11 from 19 haplogroups) were relatively rare, occurring in 1–2 out of the 6 populations under study. Two singleton haplogroups F and D were observed in Mashhad while, another two singleton haplogroups P and G were observed in the Tehran population. A Mongoloid component observed in Mashhad with the frequency of about 28.57% was represented by haplogroups M, B, and D. Thus, the data on mtDNA polymorphism indicated pronounced differentiation between western-central and eastern Iranians. Eastern Iranians (i.e. the Mashhad population) were characterized by an mtDNA pool composition similar to that of eastern and central Asia while western-central Iranian populations were characterized by an mtDNA pool composition similar to that of Europe and eastern Asia.

**Figure 4:**
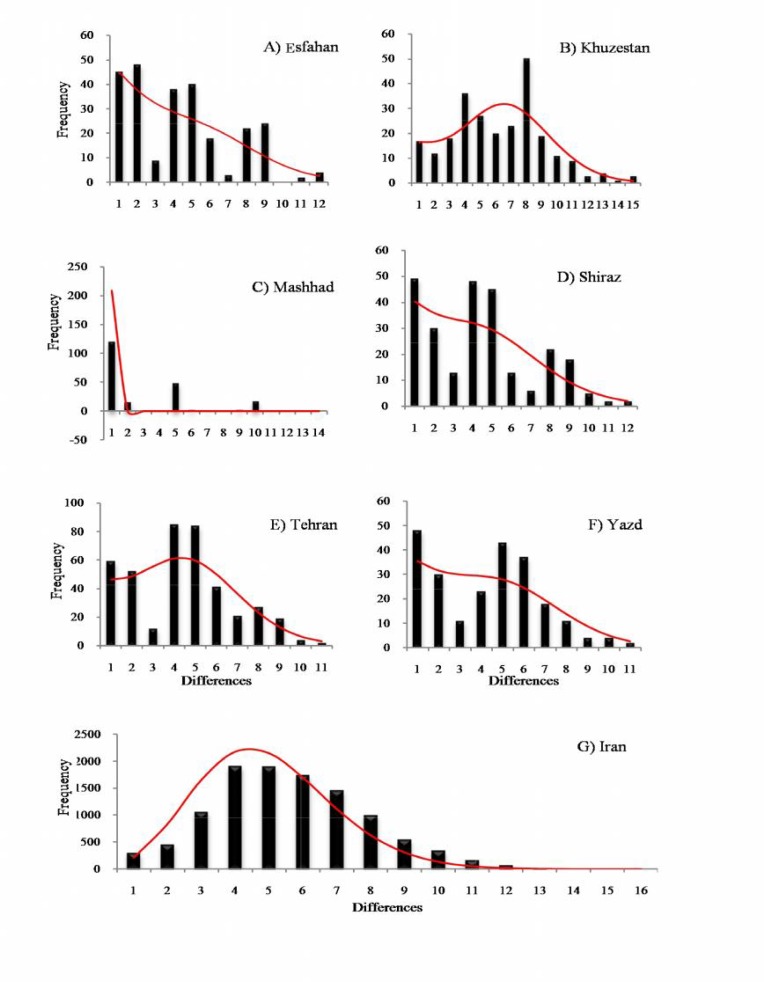
Mismatch distributions (MMD) of the Iranian populations inferred from mDNA HV2 sequences. A) Esfahan, B) Arab population from Khuzestan province, C) Mashhad D) Shiraz, E) Tehran, F) Yazd and G) Pooled samples from Iran. The observed distributions (black bars) are compared for their goodness-of-fit to a Poisson distribution under a model of sudden expansion illustrated by the thin red curves. X-axis: no. of pairwise mismatches, Y-axis; relative frequency

**Demographic analysis: **Mismatch distribution analyses (MMD) were carried out using goodness-of-fit tests based on sum of squared deviations and raggedness index (Table 3). When applied to the pooled data set of Iran, mismatch distribution was unable to reject the model of sudden expansion (P (sim ≥ obs) > 0.05) (Fig. 4G). Pooling differentiated samples, however, entail some bias; therefore we conducted the analysis population by population (Fig. 4). Mismatch curves of HV2 haplotypes were smooth and unimodal in almost all examined Iranian populations, with the exception of Mashhad (P_SSD_<0.05). Tajima's D was strongly negative and significantly different from zero for the pooled data set (D=-2.08, p<0.05) and almost all single Iranian ethnic groups with the exception of Esfahan (D=-1.18, P>0.05) and Arabs from Khuzestan province (D=-1.38, P>0.05) (Table 1). Thus, tajima’s D estimates were further confirmation for the recent expansions, which reflect an excess of singletons and low-frequency variants in the surveyed mtDNA pools resulted from recent demographic expansions. It should be noted, however, that other factors including background selection and mutation rate heterogeneity can account for deviations in these statistics [65, 66]. All these clues support the expansion model for Iran, which implies an excess of recently diverged haplotypes and a deficit of deeper coalescence events.

From the observed distribution of pairwise differences (MMD), it is possible to estimate the parameters of the theoretical model (i.e. τ) proposed by Rogers and Harpending (1992) or its simplified version [54] (Table 3). In addition, change in the effective population size can be estimated by calculating two successive values ​​of the θ includes θ_0_=2N_0_µ and θ_1_=2N_1_µ, where N_0_ and N_1_ indicate the effective population size in the past and present, respectively, and μ denotes the mutation rate of the human mtDNA D-Loop region [50] (Table 3). Time elapsed since the beginning of expansion was estimated from the equation *t *= τ/2µ, where *t *is the time since expansion and µ is the per nucleotide mutation rate multiplied by the sequence length. Estimates of the time elapsed since the beginning of expansion for the pooled data set and each Iranian population (with the exception of Mashhad) are given in Table 3. With a mutation rate of 32 % / site/ Myr [67], the τ-value of 5.66 obtained by MMD on the pooled data set is translated into an expansion time of about 30080 (55909-2338) years ago, while that of the Khuzestan province sample alone is 33343 (53084-10483). The time elapsed since the beginning of expansion for other Iranian populations ranged from 21497 (Shiraz) to 28294 (Esfahan) years ago.

**Table 3:** Basic parameters of demographic expansion for the Iranian populations inferred from HV2 segment of mtDNA sequences

**Populations**	**θ**_1_	**θ**_2_	***τ***	***t ***	**SSD (p)**	***rg*** ** (p)**
**Esfahan**	0.69	4.63	5.60 (0.17-11.92)	28294 (878-61182)	0.02 (0.58)	0.06 (0.55)
**Khuzestan province**	0.03	14.6	6.53 (2.05-10.39)	33343 (10483-53084)	0.01 (0.42)	0.03 (0.38)
**Mashhad**	0.00	447	0.00	-	0.24^*^	0.36 (0.99)
**Shiraz**	1.18	5.36	4.21 (0.52-11.49)	21497 (2670-58700)	0.02 (0.50)	0.05 (0.56)
**Tehran**	0.00	8.14	4.60 (1.30-8.00)	23488 (6643-40870)	0.02 (0.25)	0.05 (0.26)
**Yazd**	0.00	5.57	5.35 (0.02-11.56)	27318 (127-59048)	0.01 (0.57)	0.03 (0.78)
**Iran(total)**	0.01	5.22	5.66 (0.44-10.52)	30080 (2338-55909)	0.01 (0.56)	0.03 (0.58)

* p< 0.05

**Differentiation among populations:** Pairwise F_ST _genetic distances between populations were calculated and their statistical significance was estimated by 10000 permutations (Table 4). The pairwise F_ST _estimates between the Persian speaking populations including Tehran, Esfahan, Shiraz and Yazd were insignificantly low, suggesting little genetic differentiation between population pairs, which could be possibly attributable to high gene flow or the recent common origin of Persian speaking populations. In addition, Iranians, with the exception of those from Mashhad, showed high levels of differentiation with the Central Asian groups. The people from Mashhad showed lower levels of differentiation with the Azeris (-0.011) from Azerbaijan, Central Asian Kazakhs (-0.0069), Kirgizes (-0.0066) and Pathans (-0.006) from northern Pakistan. Based on the pairwise F_ST_ genetic distances, previous study shows that the Central Asian mtDNA sequences presented features between those of the Europeans and eastern Asians [68]. Several hypotheses could explain this intermediate position, but the most plausible would involve extensive levels of admixture between Europeans and eastern Asians in Central Asia.

**Figure 5:**
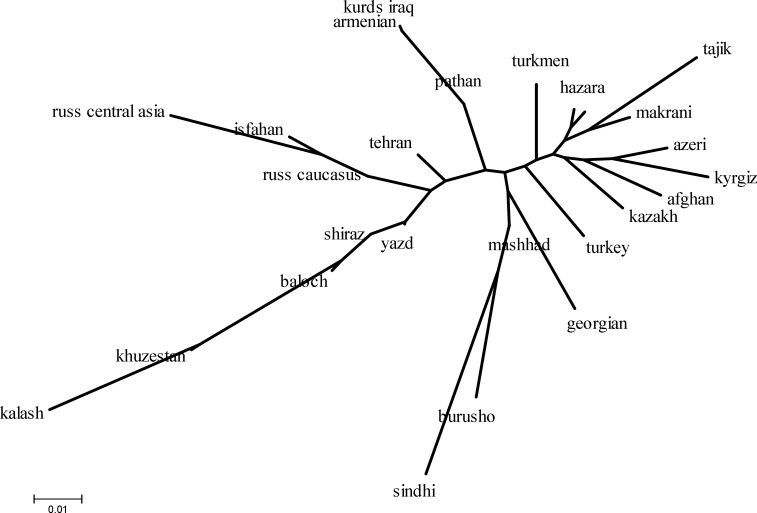
Unrooted Neighbor-joining (NJ) tree of populations based on the pairwise F_ST_ genetic distances

**Table 4:** Pairwise FST genetic distances between populations based on the mtDNA HV2 sequence

	**Ar**	**Az**	**Ge**	**Ku**	**Ru**	**Tu**	**Tu**	**Af**	**Ka**	**Ru**	**Tu**	**Ru**	**Ku**	**Ru**	**Tu**	**Tu**	**Ku**	**Ru**	**Tu**	**Tu**	**Af**	**Az**	**Ge**	**Ku**	**Ru**	**Az**	**Pa**	**Si**
**Armenians**	0																											
**Azeris**	0.016	0																										
**Georgians**	0.023	0.008	0																									
**Kurds Iraq**	-0.03	0.024	0.014	0																								
**Russ-Caucasus**	0.011	0.05	0.028	0.029	0																							
**Kurds Iraq**	0.015	0.013	0.013	0.025	0.014	0																						
**Afghan**	0.034	0.099	0.013	0.038	0.046		0																					
**Kazakh**	0.043	0.055	0.038	0.046	0.013	0.025	0.013	0																				
**Kirgiz**	0.055	0.029	0.057	0.038	0.046	0.013	0.038	0.046	0																			
**Russ- Cen.Asia**	0.008	0.028	0.038	0.046	0.013	0.038	0.046	0.038	0.046	0																		
**Tajik**	0.038	0.099	0.013	0.038	0.028	0.038	0.046	0.028	0.024	0.014	0																	
**Turkmen**	0.099		0.055	0.034	0.099	0.013	0.038	0.028	0.038	0.057	0.038	0																
**Esfahan**	0.046	0.038	0.057	0.038	0.038	0.028	0.038	0.046	0.013	0.038	0.046	0.038	0															
**Khuzestan Arabs**	0.015	0.029	0.057	0.038	0.046	0.013	0.099	0.013	0.038	0.028	0.055	0.013	0.038	0														
**Mashhad**	0.034	0.029	0.057	0.057	0.057	0.057	0.057	0.057	0.057	0.057	0.013	0.038	0.013		0													
**Tehran**	0.043	0.055	0.034	0.055	0.013	0.038	0.028	0.038	0.046	0.028	0.046	0.013	0.038	0.046	0.046	0												
**Baloch**	0.055		0.057	0.038	0.046	0.013	0.038	0.057	0.038	0.057	0.038	0.046	0.013	0.099	0.057	0.038	0											
**Burush0**	0.011	0.038	0.099	0.013	0.038	0.028	0.038	0.057	0.038	0.046	0.013	0.038	0.013	0.099	0.013	0.038	0.057	0										
**Hazara**	0.015	0.038	0.046	0.013	0.046	0.013	0.038	0.046	0.046	0.013	0.057	0.038	0.046	0.013	0.099	0.038	0.057	0.038	0									
**Kalash**	0.034	0.057	0.057	0.057	0.057	0.057	0.057	0.028	0.038	0.057	0.038	0.057	0.057	0.057	0.057	0.057	0.057	0.028	0.038	0								
**Makrani**	0.043	0.029	0.057	0.057	0.057	0.057	0.057	0.057	0.057	0.057	0.013	0.029	0.057	0.057	0.046	0.013	0.038	0.046	0.038	0.038	0							
**Makrani**	0.011	0.057	0.057	0.057	0.057	0.038	0.099	0.013	0.038	0.028	0.038	0.057	0.038	0.046	0.013	0.028	0.038	0.057	0.038	0.057	0.057	0						
**Pathan**	0.015	0.038	0.046	0.013	0.038	0.038	0.028	0.038	0.046	0.013	0.038	0.046	0.038	0.038	0.038	0.028	0.038	0.046	0.099	0.013	0.038	0.028	0					
**Sindhi**	0.034	0.046	0.038	0.038	0.038	0.038	0.028	0.038	0.046	0.013	0.038	0.046	0.038	0.038	0.038	0.028	0.038	0.028	0.038	0.057	0.038	0.057	0.057	0				
**Kalash**	0.043	0.038	0.038	0.057	0.057	0.057	0.057	0.057	0.013	0.029	0.057	0.038	0.038	0.034	0.055	0.013	0.038	0.028	0.038	0.046	0.028	0.046	0.013	0.038	0			
**Makrani**	0.055	0.038	0.038	0.038	0.046	0.013	0.038	0.046	0.038	0.038	0.038	0.038	0.099	0.013	0.038	0.028	0.038	0.057	0.038	0.046	0.013	0.038	0.013	0.099	0.013	0		
**Pathan**	0.008	0.046	0.038	0.038	0.046	0.099	0.013	0.038	0.028	0.038	0.057	0.038	0.057	0.057	0.057	0.057	0.057	0.057	0.057	0.057	0.057	0.057	0.057	0.057	0.057	0.057	0	
**Sindhi**	0.011	0.038	0.038	0.028	0.028	0.038	0.046	0.013	0.038	0.038	0.038	0.038	0.038	0.099	0.013	0.038	0.038	0.038	0.028	0.038	0.038	0.057	0.057	0.057	0.013	0.029	0.057	0

On the population level, phylogenetic tree were constructed from an F_ST_ genetic distance matrix for HV2 sequence data using the Neighbor-Joining (NJ) algorithm (Fig. 5). This clustering approach was used because it does not assume an evolutionary clock (i.e. the tree is unrooted) and produces more accurate results when closely related populations, such as human groups are analyzed [47]. Towards the top of the tree, Central Asia populations, with the exception of Russians and northwestern populations of Pakistan including Hazaras and Makranis, were placed in a single cluster, and the cluster to the left of the tree includes the remaining Iranians from Tehran, Yazd, Shiraz, Esfahan and Khuzestan province, and Russians from Caucasus and Central Asia regions (Figure 5).

**Figure 6:**
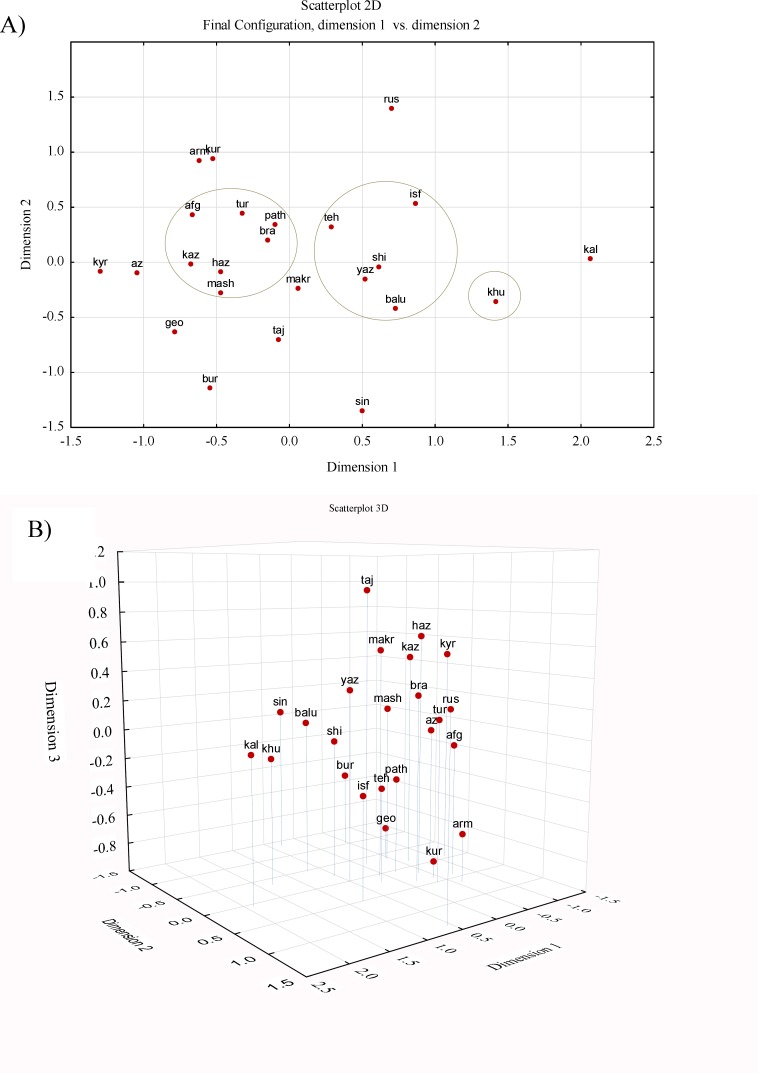
NM-MDS plot of the studied populations based on the pairwise F_ST_ genetic distances inferred from mtDNA HV2 sequence. A) 2D; and B) 3D plot

Of the Iranian populations, Mashhad is positioned closest to eastern populations, namely from Central Asia and Pakistan. A tree representation of genetic distances may be misread as a succession of evolutionary splits which is inappropriate for populations below the species level, therefore multidimensional scaling was performed on the F_ST _distance matrix for HV2 sequence data, in order to provide a visual representation of the genetic relationships in two and three-dimensional space (Figure 6). Central-western Persian speaking populations of Iran cluster together, with the dispersion being mostly in the middle of the MDS plot. As for the NJ tree, the Mashhad population was the closest to the eastern communities including those of Central Asia and Pakistan, whereas the Arab population from Khuzestan province was positioned far from other populations.

**Population structure:** The lack of a sharp geographic or ethno-linguistic structure for mtDNA HV2 sequence diversity was statistically supported by different tests. According to AMOVA results, when population samples were subdivided based on either spoken language (Iranian, Indo-Aryan, Altaic, Semitic, Armenian, Karto-Zan and Abkhazo-Adyghean language groups) or geography (pattern 1: the western (the Caucasians), the Central (the Iranians), and the eastern (the Pakistanian and Central Asian) groups; pattern 2: the western (the Caucasians), the Central (the Iranians), the southeastern (the Pakistanians), and the northeastern (the Central Asians) groups), the among-groups component of variance (i.e. Φ_CT_) was always low (Table 5). The majority of haplotype variation was indeed significantly accounted for within population differences (95.44-96.13%, p<0.001). In order to further investigate the patterns of genetic variation in geographic space, the Mantel test was used to measure the correlation between geographic and Pairwise F_ST_ genetic distance matrices. The results showed a low but significant positive correlation between the genetic and geographical distance matrices (r=0.19, P<0.05), indicating that the levels of genetic resemblance between populations is weakly dependent on geographic distances.

**Figure 7:**
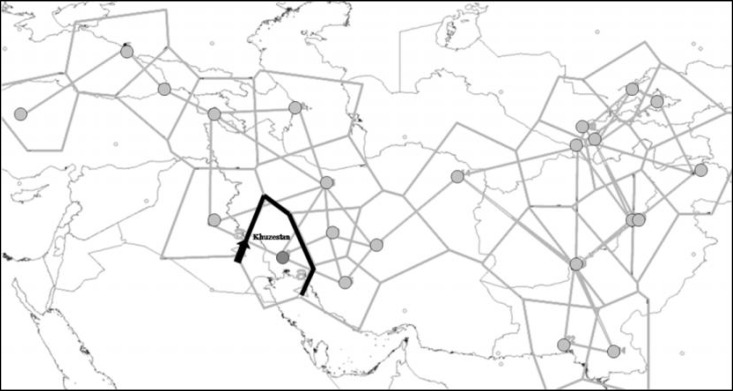
Barrier construction based on the F_ST_ genetic distances using the Monmonier’s algorithm. A genetic barrier (thick black line) was identified in a Delaunay triangulation (thin black lines

The zones of sharpest HV2 changes or putative genetic boundaries were identified using Monmonier’s algorithm based on F_ST _genetic distances. Localities were connected according to adjacency criteria, thus defining a so-called Delaunay triangulation (Fig. 7). The calculated genetic distances between populations were connected by single edges of the network. From the edges associated with the highest genetic distances, an arbitrary number of lines of maximum genetic differentiation, or genetic boundaries, were traced. The significance of each identified boundary was eventually tested by AMOVA which compares the samples on either side of that boundary. The genetic boundary inferred from HV2-based distances between populations is shown in Figure 7. A genetic barrier with the maximum degree of genetic differentiation was located between the Arab people of Khuzestan province and other Iranians. Geographical factors (i.e. residence in the southern parts of the Zagros Mountains) or limited genetic exchange due to cultural/linguistic differences are the main reasons of this differentiation.

**Table 5:** Analysis of molecular variance (AMOVA) based on the mtDNA HV2 sequence

**Pattern**	**Source of variation**	**Variance** **components**	**%**	Φ_ST_	Φ_SC_	Φ_CT_
**Geography 1**	Among groups Among populations within group Within population	0.011 0.039 1.280	0.94 3.12 95.94	0.04^*^	0.031 ^*^	0.009 ^*^
**Geography 2**	Among groups Among populations within group Within population	0.018 0.039 1.217	1.44 3.12 95.44	0.045 ^*^	0.031 ^*^	0.014 ^*^
**Linguistic**	Among groups Among populations within group Within population	0.009 0.039 1.217	0.76 3.11 96.13	0.038 ^*^	0.031 ^*^	0.007(0.1)

* p<0.05

## DISCUSSION

The Iranian populations of this study come from different regions and speak different languages**.** The populations of Esfahan, Shiraz and Khuzestan reside in geographical proximity, but speak different languages, whereas those from Mashhad, Esfahan and Shiraz speak the same language (i.e. Persian) [69] despite being located in different geographic areas. Non-significant F_ST _genetic distances for mtDNA data indicate close genetic relationships among the four Iranian populations (i.e. Tehran, Yazd, Esfahan, and Shiraz), while Mashhad and Khuzestan differ from these and from each other. The high genetic similarity of these populations suggests the existence of a single proto-Iranian population in the distant past from which greatly diverse modern Iranian ethnic groups (i.e. the Persian speaking populations) originated. The major mtDNA lineages in Iranians all exhibit a star-like network (Fig. 2) structure and unimodal mismatch distributions, which suggest the genetic effects of population expansion. The distribution of nucleotide differences between haplotypes suggests that this proto-Iranian population started to colonize Iran about 30080 years ago, which is approximately consistent with the timing of arrival and colonization of west Asia by the anatomically modern human [70]. However, additional research such as cultural practices, isotope chemistry, and mtDNA haplogroup frequencies for archaeological specimens needs to be carried out in order to confirm this scenario. Clearly, the discrepancy in mutation rates has important implications for reconstructions of human evolutionary history based on mtDNA variation, and thus caution is required when interpreting the results. The differences between estimated times regarding the beginning of colonization in west Asia by the anatomically modern human are due to the use of different mutation rates for the human mtDNA D-Loop region [67]. 

The lack of a sharp geographical or ethno-linguistic structure for mtDNA HV2 sequence diversity was statistically supported by the AMOVA test. Additionally, the Mantel test revealed a small but significant correlation (r=0.19, P<0.05) indicating that the level of genetic resemblance between populations was slightly dependent on geographic distance [62]. These results suggest that the common origin feature of the Indo-Iranian populations is obscured by the effects of gene flow from neighboring non-Indo-European populations.

Furthermore, five populations from Iran including Tehran, Yazd, Esfahan, Shiraz and Mashhad have the same linguistic origin; nevertheless, the Mashhad population is genetically different from the others, being characterized by an mtDNA pool composition similar to that of eastern and Central Asia (i.e. high frequency of haplogroups M, B and D) while the western-central Iranian populations were characterized by an mtDNA pool composition similar to that of Europe and western Asia, a finding consistent with previous studies [71]. According to pairwise F_ST_ genetic distances, the Mashhad population showed a close genetic relationship with the Central Asian populations; suggesting the presence of a gene flow from these populations. Central Asian mtDNA sequences presented features between European and eastern Asian sequences [68] in several parameters such as frequencies of certain nucleotides, levels of nucleotide diversity, mean number of pairwise differences, and pairwise F_ST _genetic distances. Several hypotheses could explain the intermediate position of Central Asia between Europe and the eastern Asia, but the most plausible would involve extensive levels of admixture between the Europeans and eastern Asians in Central Asia, possibly enhanced during the Silk Road trade and clearly after eastern and the western Eurasian human groups had diverged [72]. In addition, the lowest diversity among the Iranians, belonged to the Mashhad population (*h*= 0.428), which indicates the effect of evolutionary forces such as inbreeding, drift or bottleneck events during the evolution of this population.

Furthermore, Yazd, Shiraz, Esfahan and Khuzestan province populations reside in geographical proximity, but the Arab population of Khuzestan is genetically distant from the others, reflecting their different origin. The Persian language belongs to the Iranian branch of the Indo-European family, whereas the Arabic language spoken in parts of Khuzestan province belongs to the Semitic family [69]. In the present study, using Monmonier’s maximum differences algorithm [57] based on the F_ST_ genetic distances, we showed differentiation among the Arab people of Khuzestan province and other Iranians. Geography [73] and cultural/linguistic differences due to inbreeding or genetic exchange limited to other Arabic speaking populations in the south west of Iran, [74, 75] can be the main reasons of this differentiation.

## References

[1] Luis JR, Rowold DJ, Regueiro M, Caeiro B, Cinnioğlu C, Roseman C, Underhill PA, Cavalli-Sforza LL, Herrera RJ (2004). The levant versus the horn of africa: Evidence for bidirectional corridors of human migrations. Am J Hum Gen.

[2] Farjadian S, Sazzini M, Tofanelli S, Castrì L, Taglioli L, Pettener D, Ghaderi A, Romeo G, Luiselli D (2011). Discordant patterns of mtdna and ethno-linguistic variation in 14 iranian ethnic groups. Hum Hered.

[3] Weng Z, Sokal RR (1995). Origins of indo-europeans and the spread of agriculture in europe: Comparison of lexicostatistical and genetic evidence. Hum Biol.

[4] Gimbutas M (1973). Old europe c. 7000-3500 bc: The earliest european civilization before the infiltration of the indo-european peoples. J IndoEu Stud.

[5] Gimbutas M (1977). The first wave of eurasian pastoralists into copper age europe. J Indo-Eur Stud.

[6] Gimbutas M (1980). The kurgan wave 2 (c 3400-3200 bc) into europe and the following transformation of culture in the transformation of european and anatolian culture c 4500-2500b C And its legacy, partii. J Indo-Eur Stud.

[7] Mallory JP, Adams DQ (2006). The oxford introduction to proto-indo-european and the proto-indo-european world.

[8] Moorjani P, Thangaraj K, Patterson N, Lipson M, Loh P-R, Govindaraj P, Berger B, Reich D, Singh L (2013). Genetic evidence for recent population mixture in india. Am J Hum Gen.

[9] Renfrew C (1990). Archaeology and language: The puzzle of indo-european origins.

[10] Menozzi P, Piazza A, Cavalli-Sforza L (1978). Synthetic maps of human gene frequencies in europeans. Sci.

[11] Sokal RR, Menozzi P (1982). Spatial autocorrelations of hla frequencies in europe support demic diffusion of early farmers. Am Nat.

[12] Sokal RR, Oden NL, Thomson BA (1992). Origins of the indo-europeans: Genetic evidence. Proc Natl Acad Sci USA.

[13] Sokal RR, Oden NL, Wilson C (1991). Genetic evidence for the spread of agriculture in europe by demic diffusion. Nature.

[14] Drummond AJ, Rambaut A, Shapiro B, Pybus OG (2005). Bayesian coalescent inference of past population dynamics from molecular sequences. Mol Biol Evol.

[15] Ho SY, Shapiro B (2011). Skyline‐plot methods for estimating demographic history from nucleotide sequences. Mol Ecol Resour.

[16] Campos PF, Willerslev E, Sher A, Orlando L, Axelsson E, Tikhonov A, Aaris-Sørensen K, Greenwood AD, Kahlke R-D, Kosintsev P (2010). Ancient DNA analyses exclude humans as the driving force behind late pleistocene musk ox (ovibos moschatus) population dynamics. Proc Natl Acad Sci USA.

[17] Finlay EK, Gaillard C, Vahidi S, Mirhoseini S, Jianlin H, Qi X, El-Barody M, Baird J, Healy B, Bradley DG (2007). Bayesian inference of population expansions in domestic bovines. Biol Lett.

[18] Atkinson QD, Gray RD, Drummond AJ (2008). Mtdna variation predicts population size in humans and reveals a major southern asian chapter in human prehistory. Mol Biol Evol.

[19] Stiller M, Baryshnikov G, Bocherens H, d'Anglade AG, Hilpert B, Münzel SC, Pinhasi R, Rabeder G, Rosendahl W, Trinkaus E (2010). Withering away—25,000 years of genetic decline preceded cave bear extinction. Mol Biol Evol.

[20] Kitchen A, Miyamoto MM, Mulligan CJ (2008). Utility of DNA viruses for studying human host history: Case study of jc virus. Mol Phylogenet Evol.

[21] Magiorkinis G, Magiorkinis E, Paraskevis D, Ho SY, Shapiro B, Pybus OG, Allain J-P, Hatzakis A (2009). The global spread of hepatitis c virus 1a and 1b: A phylodynamic and phylogeographic analysis. PLoS Med.

[22] Avise JC (2000). Phylogeography: The history and formation of species.

[23] Cavalli-Sforza LLL, Menozzi P, Piazza A (1994). The history and geography of human genes.

[24] Chunjie X, Cavalli-Sforza L, Minch E, Ruofu D (2000). Principal component analysis of gene frequencies of chinese populations. Sci China Ser B.

[25] Du R, Xiao C, Cavalli-Sforza L (1997). Genetic distances between chinese populations calculated on gene frequencies of 38 loci. Sci China C Life Sci.

[26] Quintana-Murci L, Chaix R, Wells RS, Behar DM, Sayar H, Scozzari R, Rengo C, Al-Zahery N, Semino O, Santachiara-Benerecetti AS (2004). Where west meets east: The complex mtdna landscape of the southwest and central asian corridor. Am J Hum Gen.

[27] Shepard E, Herrera R (2006). Iranian str variation at the fringes of biogeographical demarcation. Forensic Sci Int.

[28] Regueiro M, Cadenas A, Gayden T, Underhill P, Herrera R (2006). Iran: Tricontinental nexus for y-chromosome driven migration. Hum Hered.

[29] Farjadian S, Ghaderi A (2007). Hla class ii genetic diversity in arabs and jews of iran. Iran J Immunol.

[30] Farjadian S, Ghaderi A (2007). Hla class ii similarities in iranian kurds and azeris. Int J Immunogenet.

[31] Farjadian S, Ota M, Inoko H, Ghaderi A (2009). The genetic relationship among iranian ethnic groups: An anthropological view based on hla class ii gene polymorphism. Mol Biol Rep.

[32] Schönberg A, Theunert C, Li M, Stoneking M, Nasidze I (2011). High-throughput sequencing of complete human mtdna genomes from the caucasus and west asia: High diversity and demographic inferences. Eur J Hum Genet.

[33] Irwin JA, Ikramov A, Saunier J, Bodner M, Amory S, Röck A, O’Callaghan J, Nuritdinov A, Atakhodjaev S, Mukhamedov R (2010). The mtdna composition of uzbekistan: A microcosm of central asian patterns. Int J Legal Med.

[34] Al-Zahery N, Saunier J, Ellingson K, Parson W, Parsons TJ, Irwin JA (2013). Characterization of mitochondrial DNA control region lineages in iraq. Int J Legal Med.

[35] Kumar S, Nei M, Dudley J, Tamura K (2008). Mega: A biologist-centric software for evolutionary analysis of DNA and protein sequences. Brief Bioinform.

[36] Tajima F (1983). Evolutionary relationship of DNA sequences in finite populations. Genetics.

[37] Nei M (1987). Molecular evolutionary genetics.

[38] Excoffier L, Smouse PE, Quattro JM (1992). Analysis of molecular variance inferred from metric distances among DNA haplotypes: Application to human mitochondrial DNA restriction data. Genetics.

[39] Andrews RM, Kubacka I, Chinnery PF, Lightowlers RN, Turnbull DM, Howell N (1999). Reanalysis and revision of the cambridge reference sequence for human mitochondrial DNA. Nat Genet.

[40] Van Oven M, Kayser M (2009). Updated comprehensive phylogenetic tree of global human mitochondrial DNA variation. Hum Mutat.

[41] Bryant D, Moulton V (2004). Neighbor-net: An agglomerative method for the construction of phylogenetic networks. Mol Biol Evol.

[42] Huson DH, Bryant D (2006). Application of phylogenetic networks in evolutionary studies. Mol Biol Evol.

[43] Kayser M, Roewer L, Hedman M, Henke L, Henke J, Brauer S, Krüger C, Krawczak M, Nagy M, Dobosz T (2000). Characteristics and frequency of germline mutations at microsatellite loci from the human y chromosome, as revealed by direct observation in father/son pairs. Am J Hum Gen.

[44] Sigurðardottir S, Helgason A, Gulcher JR, Stefansson K, Donnelly P (2000). The mutation rate in the human mtdna control region. Am J Hum Gen.

[45] Walker A, Smith S, Smith S (1987). Mitochondrial DNA and human evolution. Nat..

[46] Posada D (2008). Jmodeltest: Phylogenetic model averaging. Mol Biol Evol.

[47] Saitou N, Nei M (1987). The neighbor-joining method: A new method for reconstructing phylogenetic trees. Mol Biol Evol.

[48] Kruskal JB (1964). Nonmetric multidimensional scaling: A numerical method. Psychometrika.

[49] Tajima F (1989). Statistical method for testing the neutral mutation hypothesis by DNA polymorphism. Genetics.

[50] Schneider S, Excoffier L (1999). Estimation of past demographic parameters from the distribution of pairwise differences when the mutation rates vary among sites: Application to human mitochondrial DNA. Genetics.

[51] Rogers AR, Harpending H (1992). Population growth makes waves in the distribution of pairwise genetic differences. Mol Biol Evol.

[52] Slatkin M, Hudson RR (1991). Pairwise comparisons of mitochondrial DNA sequences in stable and exponentially growing populations. Genetics.

[53] Excoffier L (2004). Patterns of DNA sequence diversity and genetic structure after a range expansion: Lessons from the infinite‐island model. Mol Ecol.

[54] Harpending H (1994). Signature of ancient population growth in a low-resolution mitochondrial DNA mismatch distribution. Hum Biol.

[55] Li W-H (1977). Distribution of nucleotide differences between two randomly chosen cistrons in a finite population. Genetics.

[56] Stenico M, Nigro L, Barbujani G (1998). Mitochondrial lineages in ladin–speaking communities of the eastern alps. Proc R Soc Lond B Biol Sci.

[57] Monmonier MS (1973). Maximum‐difference barriers: An alternative numerical regionalization method. Geogr Anal.

[58] Bosch E, Calafell F, Pérez-Lezaun A, Comas D, Mateu E, Bertranpetit J (1997). Population history of north africa: Evidence from classical genetic markers. Hum Biol.

[59] Manni F, Guerard E, Heyer E (2004). Geographic patterns of (genetic, morphologic, linguistic) variation: How barriers can be detected by using monmonier's algorithm. Hum Biol.

[60] Slatkin M (1995). A measure of population subdivision based on microsatellite allele frequencies. Genetics.

[61] Wright S (1965). The interpretation of population structure by f-statistics with special regard to systems of mating. Evol.

[62] Mantel N (1967). The detection of disease clustering and a generalized regression approach. Cancer Res.

[63] Dixon P (2003). Vegan, a package of r functions for community ecology. J Veg Sci.

[64] Ersts P Geographic distance matrix generator software. Version 1.2. 3. American museum of natural history. Center for Biodiversity and Conservation.

[65] Tajima F Measurement of DNA polymorphism. Mech Mol Evol.

[66] Aris-Brosou S, Excoffier L (1996). The impact of population expansion and mutation rate heterogeneity on DNA sequence polymorphism. Mol Biol Evol.

[67] Billot C, Engel CR, Rousvoal S, Kloareg B, Valero M (2003). Current patterns, habitat discontinuities and population genetic structure: The case of the kelp laminaria digitata in the english channel. Mar Ecol Prog Ser.

[68] Comas D, Calafell F, Mateu E, Pérez-Lezaun A, Bosch E, Martínez-Arias R, Clarimon J, Facchini F, Fiori G, Luiselli D (1998). Trading genes along the silk road: Mtdna sequences and the origin of central asian populations. Am J Hum Gen.

[69] Grimes BF, Grimes JE (2000). Linguistics SIo. Ethnologue.

[70] Maca-Meyer N, González AM, Larruga JM, Flores C, Cabrera VM (2001). Major genomic mitochondrial lineages delineate early human expansions. BMC Genet.

[71] Houshmand M, Sanati M-H, Vakilian M, Akuchekian M, Babrzadeh F, Teimori M, Farhud D (2004). Investigation of the mitochondrial haplogroups m, bm, n, j, k and their frequencies in five regions in iran. Iranian J Biotechnol.

[72] Comas D, Plaza S, Wells RS, Yuldaseva N, Lao O, Calafell F, Bertranpetit J (2004). Admixture, migrations, and dispersals in central asia: Evidence from maternal DNA lineages. Eur J Hum Genet.

[73] Barbujani G, Sokal R (1991). Genetic population structure of italy. Ii. Physical and cultural barriers to gene flow. Am J Hum Genet.

[74] Barbujani G, Sokal RR (1990). Zones of sharp genetic change in europe are also linguistic boundaries. Proc Natl Acad Sci USA.

[75] Zei G, Barbujani G, Lisa A, Fiorani O, Menozzi P, Siri E, Cavalli‐Sforza LL (1993). Barriers to gene flow estimated by surname distribution in italy. Ann Hum Genet.

